# Driving-Related Cognitive Abilities: Evaluating Change over Time in a Sample of Older Adults Undergoing an Assessment Regarding Fitness to Drive

**DOI:** 10.3390/ijerph191912806

**Published:** 2022-10-06

**Authors:** Stefania Balzarotti, Eleonora Pagani, Ilaria Telazzi, Martina Gnerre, Federica Biassoni

**Affiliations:** Department of Psychology, Catholic University of the Sacred Heart, Largo Gemelli 1, 20123 Milan, Italy

**Keywords:** driving-related abilities, cognitive change, aging, restricted drivers

## Abstract

Advancing age can bring a decline in many driving-related cognitive abilities. For this reason, public safety concern has raised about older adults’ driving performance, and many countries have adopted screening polices to assess older drivers’ fitness to drive. As a result of such assessments, authorities may impose behavioral restrictions to driving. The present study examines whether driving-related cognitive abilities change over time and compares drivers either restricted or not by licensing authorities after the first assessment. The data were derived from a database provided by a service of psychodiagnostic assessment of fitness to drive. This database contained data of people referred for cognitive assessment in order to renew their driving license over the period of 2016 to 2022. The sample included 58 cognitively healthy old drivers (mean age = 82.79, SD = 6.13; 97% men) with a follow-up examination (T2) after a period ranging from one to four years (M = 1.59, SD = 0.72) since the first assessment. Cognitive assessments were conducted using the standard test battery from the Vienna Test System (VTS8; ©Schuhfried GmbH, Mödling, Austria). Decision time variability, motor time, reaction time under stress, and obtaining an overview did not show significant changes between T1 and T2, whereas selective attention and inductive reasoning significantly decreased over time in both groups. Improvements in processing speed consistent with practice effects emerged at T2. Restricted drivers (*n* = 41) maintained significantly worse performances than unrestricted drivers (*n* = 17) in the follow-up assessment. Chronological age was associated with higher reaction time under stress, while education showed a buffering role against a decrease in perceptual speed. Overall, although older drivers’ driving-related cognitive abilities remain relatively stable over the short-term, the decline in some cognitive functions deserves reevaluation and monitoring.

## 1. Introduction

It is well-known that population aging is one of the main current and future societal challenges. The global population of adults over the age 65 of is estimated to rise from 10% in 2022 to 16% in 2050, being more than twice the number of children aged 5 or less [1]. Because of the health and socioeconomic consequences of this demographic transformation, in the last decades, research has grown addressing aging-related processes and diseases [2]. Within psychological research, numerous studies have addressed cognitive aging, showing that a decline in cognitive functions is associated with both normal (or “healthy”) and pathological aging, even though with significant individual variability (e.g., [3,4]; for a review, see [5,6]).

Among the implications that cognitive aging may have for various domains of everyday activities that are important to older adults, attention has been given to driving behavior [7]. Driving a motor vehicle is a complex activity that involves sensory, cognitive, and higher executive functions, and various studies have shown that advancing age can bring a decline in many driving-related cognitive abilities [8,9,10]. Several studies have examined older drivers’ accident rate per miles driven, and it is generally agreed that this rate is higher than among other age groups, except for the youngest drivers [11,12,13,14,15] (for exceptions, see [16,17,18]). For this reason, public safety concern has raised about older adults’ fitness to drive and their greater chance to be involved in car crashes [19]. In addition, many countries worldwide have adopted screening polices for elderly drivers to identify at-risk drivers [20,21,22].

At the same time, maintaining a driving license entails many benefits for older adults, with a significant impact on their quality of life and wellbeing [23]. Driving cessation has been associated with a higher risk of depressive symptoms, admission in long-term care facilities, and mortality (e.g., [24,25]; for a review, see [26]). Rather than suspension, driver licensing agencies may thus impose behavioral restrictions, such as driving only in daylight, avoiding some road types (e.g., highways), speed limitations, and proximity to home. The purpose is to preserve mobility for older drivers with some functional impairment and simultaneously to reduce their crash risk [27].

Thus far, several studies have examined the relationship between cognitive functions, driving performance, and age (e.g., [28,29,30]; for a review see [31]). By using methods such as cognitive test batteries, on-road driving tests, or driving simulator performance, this research has shown age-related decline in both cognitive and driving performance. In addition, these studies have tried to identify the cognitive abilities that are the most essential predictors of driving competence (e.g., processing speed, executive functions, spatial ability, working memory) [32]. Notably—although researchers agree that driving-related cognitive abilities decline with age—it has been suggested that chronological age should not be used as an index of driving competence. First, research has consistently shown that cognitive abilities independently explain a significant amount of variance in driving performance [33,34]. Second, there are large individual differences in cognitive abilities even at older ages [32,35]. These results provide evidence in support of a role for the assessment of cognitive functions at an individual level as a more accurate measure of driving ability than chronological age alone [21,32].

So far, existing research mostly consists of cross-sectional studies, whereas less research has employed a longitudinal design to examine changes in older drivers’ driving-related cognitive abilities and/or driving performance over time. Nonetheless, longitudinal data may provide important information concerning cognitive trajectories to determine whether driving-related cognitive abilities should be monitored and how to time follow-up assessments. A group of prospective studies [36,37,38] have focused on older drivers with brain pathology (e.g., dementia and Parkinson disease) in an attempt to identify the progression of driving impairment over time. Drivers with neurological disease showed a poorer cognitive and driving performance than controls at baseline. In addition, a more profound decline in driving performance emerged for drivers with a neurological condition than for controls over time (i.e., after 2–3 years). Most relevant to the present study, a three-year longitudinal study employing a sample of healthy older adults [39] found that longitudinal changes in the mean levels of driving performance (i.e., safety errors in on-road driving tests) were small from year to year. Likewise, little changes or even small improvements emerged in older adults’ performance in tests measuring driving-related cognitive abilities. Overall, the authors concluded that normative aging-related declines in driving performance emerge slowly.

Relatedly, some studies examining the longitudinal pattern of performance on neuropsychological cognitive tests in healthy older adults have failed to observe the expected age-related decline. For instance, a recent study examining older adults’ performance on the Montreal Cognitive Assessment (MoCA) over a four-year period [40] found that participants’ performance increased after 12 months of testing and then remained stable until 48 months. This improvement between the first and the second assessment was mainly observed in those individuals that scored lower at baseline, and was interpreted as a consequence of practice effects. Practice effects comprise factors such as memory for test items, procedural learning, and general experience with testing, and typically lead to a better performance at the time of the second assessment as a result of repeated exposure to the same test materials [41,42]. A recent metanalysis estimated the practice effects on several cognitive tests that are often employed in neuropsychological assessment, showing that the magnitude of these effects is moderated by variables such as age, cognitive domain, and length of the test–retest interval [42].

Finally, little research has examined the differences between restricted and unrestricted older drivers. Most studies have compared accident rates in an attempt to determine whether restrictions represent an effective tool for mitigating the number of crashes per year and whether they can prolong the period of crash-free driving for older drivers [27,43] (for a review see [44]). One study [35] compared restricted and unrestricted drivers’ performance at cognitive tests, finding that restricted drivers scored significantly lower than the other drivers. Although restrictions are usually imposed in an attempt to better match the demands of the driving task to the driver’s capacity to drive safely [27], they can also lead to reduced driving practice, which has been linked to a higher crash risk [16,17,18].

### The Present Study

The first goal of the present study is to examine whether the driving-related cognitive abilities of older drivers referred for cognitive assessment change over time. In more detail, the study considers older drivers’ performance on cognitive tests at two time points (i.e., first assessment and follow-up) and examines how performance on the Drivesta test battery of the Vienna Test System (VTS8; ©Schuhfried GmbH; [45]) changes from the first to the second administration. In Italy, the Provincial Medical Commissions of Public Health Services are in charge of regular assessment of medical fitness to drive for drivers with specific types of pathologies (e.g., heart disease, diabetes, and brain disease). These commissions may also refer the driver for cognitive assessment. Based on the results of these assessments, medical commissions may either renew or suspend the driving license. In the case of renewal, medical commissions may impose behavioral restrictions and establish how long the license is valid (after this period, the driver needs to be reevaluated).

Second, we compare drivers either restricted or not by licensing authorities after the first assessment in order to test the hypothesis that restricted drivers may show a worsened performance at T2 compared with the unrestricted drivers.

## 2. Materials and Methods

### 2.1. Participants

The data used in this study were derived from a database provided by the service of psychodiagnostic assessment of fitness to drive of the Catholic University of the Sacred Heart, Milan, Italy. The database contained data of people referred for cognitive assessment by the Provincial Medical Commissions of Public Health Services in Milan in order to obtain their driving license renewed over the period of 2016–2022.

In more detail, the data to be used in this study were extracted from the database considering those people who had been assessed at least two times: a first assessment (T1) and a follow-up examination (T2). Other inclusion criteria were (a) age over 65, (b) being cognitively healthy (i.e., no diagnosis of either cognitive impairment or brain disease), (c) no diagnosis of psychiatric disorders, (d) no sleep disorders, and (e) no reported use of benzodiazepines or antidepressant medication. This led to a sample of 58 older adults (mean age = 82.79, SD = 6.13, range 69–93; 97% male) assessed after a period ranging from one to four years (M = 1.59, SD = 0.72) since the first assessment.

**Demographic information**. Of our sample, 79% were married, 2% divorced, 12% widow, and 8% single. Concerning education, 2% of the sample accomplished less than five years of school, 31% reported to have accomplished primary school (5 years), 17% had up to 8 years of education, 38% up to 13 years, and 12% reported to have attended school for more than 13 years. In addition, 97% of the sample was retired.

**Health status**. The participants reported chronic conditions that are common in older age, such as heart disease (43%), high blood pressure (30%), diabetes (9%), hearing prosthetic devices (20%), cataract correction surgery (15%), and dyslipidemia (7%).

**Driving behavior and habits**. Few drivers (12%) reported to have been involved in car accidents over the past five years. Most of our sample (97%) reported to drive regularly (at least twice a week) along urban routes, but a significant percentage also reported driving along suburban routes (71%) as well as on the highway (47%). Driving a car was reported to be the main means of transportation in order to accomplish the needs of daily living.

### 2.2. Measures

**Restrictions to driving.** Here, 15% of drivers (*n* = 9) reported current restrictions to driving imposed by authorities (i.e., driving in highway or at night not permitted, or driving permitted only within a certain distance of home) at T1. At T2, the percentage increased to 70% (*n* = 41). 

**Standardized Assessment of Driving-related Cognitive Abilities.** The assessment included four tests from the computerized test battery of the Vienna Test System (VTS8; ©Schuhfried GmbH; [45]) and a paper–pencil test to assess inductive reasoning (Raven’s colored matrices). Significant correlations have been shown between the scores obtained at the VTS tests and driving performance in on-road tests [46].

**Reaction time (Decision and Motor Time).** In the reaction test (RT), the respondent was instructed to react to a critical stimulus combination (acoustic signal and visual stimulus). Notably, reaction times were measured using a rest and a response button, allowing for discrimination between decisions and motor time. The mean decision time (DT) is computed as the interval between the onset of the target stimulus to the lifting of the finger from the rest button, while physical motor time (MT) is measured by the latency from the start of the finger-lifting movement to the moment when the response key is pressed. Three indices were derived: mean DT, mean MT, and DT standard deviation (DTSD). As a result of technical problems, data of the RT were not available for two participants.

**Reactivity under sensory stress.** The determination test requires to quickly respond to rapidly changing visual and auditory stimuli, thus tapping the abilities of cognitive shifting and flexibility. The test is administered so that the stimuli are presented a little faster than would be optimal for the respondent’s reaction speed. The score corresponds to the number of correct responses. Due to technical problems, data of the DT were not available for two participants.

**Selective attention and concentration**. In the cognition (COG) test, the respondent’s task is to determine whether an abstract target figure matches one of four comparison figures. The respondent must press the green button on the response panel if the figures match or the red button if they don’t. The score consists in the average time of correct rejections. 

**Obtaining an overview.** The Adaptive Tachistoscopic Traffic Perception Test (ATAVT) assessed observational ability by briefly (<1 s) presenting pictures of traffic situations. After viewing the picture, the respondent was asked to identify whether the picture included pedestrians, vehicles, bicycles/motorbikes, road signals, and traffic lights. The score (estimated according to the Rasch model) represents the number of items for which all visible object classes were correctly identified by the respondent and if no object class that was not visible in the traffic scene was marked.

**Inductive reasoning.** Raven’s Colored Progressive Matrices [47,48] were used to evaluate inductive reasoning. The test consisted of 36 visual matrices of ascending difficulty. The respondent’s task was to identify the rules that govern each matrix and to fill out the empty space by selecting the correct answer from eight alternatives. The total raw score range was between 0–36.

### 2.3. Procedure

At both T1 and T2, the assessments were conducted by a traffic psychologist according to the following procedure. First, participants were asked to read and sign written informed consent and GDPR-compliant privacy forms. They were also asked to give their consent if they agreed that their data would be used for research purposes in an aggregate and de-identified way. Second, a clinical interview took place to collect anamnestic information including health status and driving behavior. Then, the psychologist administered the cognitive tests, including Raven’s Colored Progressive Matrices [47,48] and other tests from the Vienna Test System (©Schuhfried GmbH). The psychologist explained the instructions for all the tests to the participants. The whole procedure lasted on average two hours.

In the follow-up assessment (T2), the Provincial Medical Commissions of Public Health Services could refer drivers for cognitive assessment, either asking for a complete evaluation (i.e., including all the tests administered at T1) or for a briefer assessment including three out of five cognitive tests. For this reason, the scores of the tests assessing selective attention and inductive reasoning were not available for 13 participants.

### 2.4. Study Design

The study employed pre-existing, archival data. The source database contained approximately 2000 evaluations of fitness to drive conducted over the period 2016–2022. The researchers inspected this information and extracted the data used in this study based on the inclusion criteria described above. After extraction, all data were deidentified.

### 2.5. Data Analysis

Repeated measures Analysis of Covariance (ANCOVA) was used to test whether the performances at cognitive tests differed between T1 and T2. Current restrictions to driving at T2 (yes/no) were included as a fixed factor. Age, years of education, and the length of time interval between T1 and T2 were included as covariates. Following Schneider et al. [49], covariates were mean centered. Moreover, ANCOVAs were used to estimate the within*covariate interactions, while a standard repeated measure ANOVA was used to evaluate any effects not involving the covariates. As the two groups (with and without restrictions) had different sample sizes, Levene’s test was used to examine the equality of variances. Finally, significant interaction effects were probed using MEMORE macro for SPSS [50]. In more detail, we used Model 2, bias-corrected bootstrap CI, bootstrap 5000 samples, and the Johnson–Neyman procedure for the conditional effects.

Each score obtained in the cognitive tests was used as a dependent variable. T-scores were examined when available in the VTS software, as they compare the performances of our sample with those of a normative sample.

## 3. Results

The descriptive statistics are shown in Table 1, while the results of the ANCOVAs are displayed in Table 2. Significant effects are reported in bold, while marginally significant (*p* < 0.10) effects are in italics.

Concerning change over time, the results showed that decision time improved at T2, while selective attention and inductive reasoning significantly decreased over time. No significant changes between T1 and T2 emerged for decision time variability, motor time, reaction time under stress, and obtaining an overview.

Concerning restrictions to driving, the main effect of this variable was significant for almost all cognitive abilities (the effect was marginally significant for motor time). The *t*-test showed that drivers reporting restrictions to driving imposed by the authorities after T1 assessment obtained significantly poorer performances also at the T2 assessment, with the exception being for obtaining an overview, *t*(70) = 0.61, *p* = 0.545. This was due to the decreased performance of unrestricted drivers at T2. The interaction Time × Restriction was never significant.

Concerning the covariates, age was positively associated with reaction time under stress (i.e., increasing age was related to a higher reaction time), education years was positively associated with inductive reasoning, while the length of the interval between T1 and T2 assessments was negatively associated with reaction time under stress and inductive reasoning.

**Figure 1:**
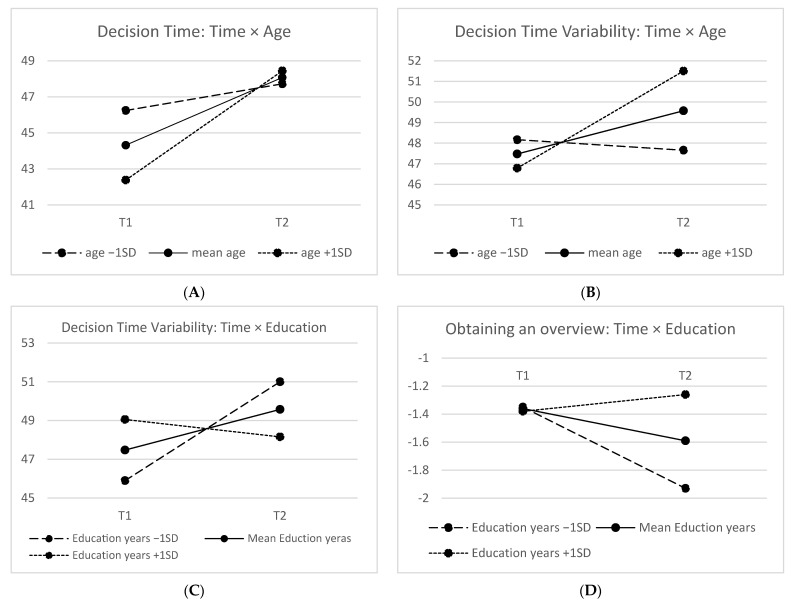

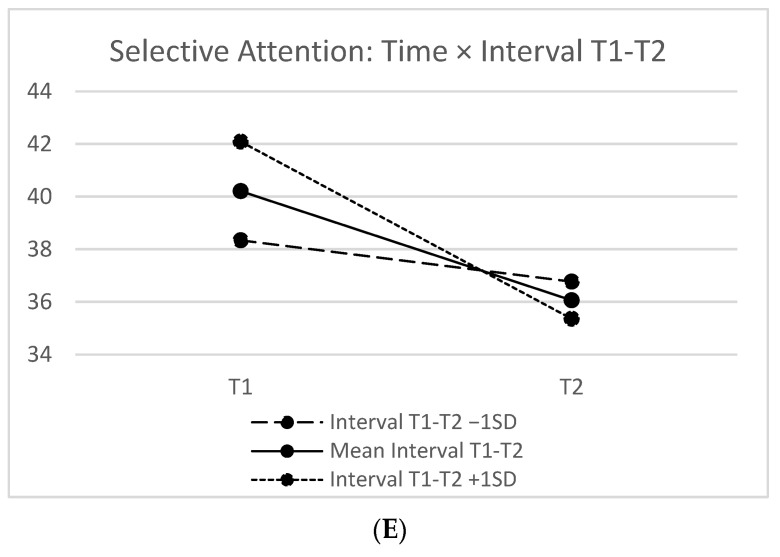
Significant interaction effects: (**A**) the effect of time on decision time by age; (**B**) the effect of time on decision time variability by age; (**C**) the effect of time on decision time variability by education; (**D**) the effect of time on perceptual speed by education; (**E**) the effect of time on selective attention by T1–T2 interval length.

Finally, some significant interaction effects emerged between time and the covariates (see Figure 1). First, the effect of time on decision time was moderated by age: Improvements at T2 were larger (and significant) for older drivers (age >79). Second, the effect of time on decision time variability was moderated by age and education—improvements were significant for older drivers (age >84) with lower education (years < 9). Third, the effect of time on the perceptual ability to obtaining an overview was moderated by education—a decrease at T2 was larger for drivers with a lower education. Finally, the effect of time on selective attention was moderated by the length of the T1–T2 interval—the decrease at T2 was larger when the interval was longer.

## 4. Discussion

In this study, we examined whether the driving-related cognitive abilities of older drivers referred for cognitive assessment change over time. To the best of our knowledge, few studies so far have provided longitudinal evidence concerning older drivers’ driving-related cognitive abilities [39], and most of these prospective studies have concerned drivers with some brain pathology (e.g., dementia) in an attempt to identify the progression of driving impairment over time [36,37,38]. Moreover, we compared restricted and unrestricted drivers’ performances at T2 to examine whether the two groups showed different trajectories over time.

First, the results showed that most of the tested cognitive abilities were stable: No significant changes between T1 and T2 emerged in decision time variability, motor time, reaction time under stress, and obtaining an overview. These results are consistent with those obtained by a previous three-year longitudinal study [39], and seem to suggest that older drivers’ driving-related cognitive abilities remain relatively stable over the short-term. However, we also found that selective attention and inductive reasoning significantly decreased in both groups, supporting the need for reevaluation and monitoring. 

Surprisingly, decision time improved at T2, and this improvement was larger for drivers aged >79. These drivers scored significantly lower than other drivers at the T1 assessment. This result was unexpected. Decision time can be considered a measure of processing speed [51], which has been considered as a cognitive “primitive” underlying higher cognitive abilities [52]. A possible explanation is that this improvement was due to practice effects on performance, as the same tests were used in the T1 and T2 assessments. Previous prospective studies have found similar improvements in older adult’s cognitive performance over repeated assessments [39,40]. A low baseline performance and subsequent improvement have been interpreted as a result of practice effects [40]. In the same vein, the results also showed that drivers aged >84 and with lower levels of education improved in decision time variability at T2. 

An open question concerns the reason practice effects have not occurred for all the cognitive abilities tested (selective attention and inductive reasoning significantly decreased over time). The existing literature suggests that gains for tests in some cognitive domains (e.g., processing speed and executive functions) may be higher than for tests in other domains (e.g., visuospatial abilities). Moreover, a high degree of within-domain variability exists, so that if practice effects are found regarding performance to a specific test, this result cannot be generalized to other tests measuring the same cognitive function. In other words, some tests may be more susceptible to practice effects than others. Concerning Go/No Go reaction tasks, it has been shown that repeated practice can lead to faster sensorimotor processing and to a reduced response time [53]. Nonetheless, research has not yet examined whether test batteries assessing fitness to drive, such as the VTS, may be prone to practice effects.

Concerning driving restrictions, we found that restricted drivers after T1 assessment also obtained significantly poorer performances than unrestricted drivers at T2 assessment. However, the interaction effect Time × Restriction was never significant, indicating that the two groups maintained similar levels of performance at T1 and T2 (i.e., restricted drivers did not show a decline compared to unrestricted drivers). This result adds to the findings of a previous, cross-sectional study showing significant differences in cognitive performance between restricted and unrestricted drivers [35]. An exception was represented by the perceptual ability to obtain an overview (or perceptual speed). In this case, performances at T2 did not differ between restricted and unrestricted drivers, as these latter showed a performance drop at T2.

Finally, our results showed that education was positively related to inductive reasoning and moderated the effect of time on the perceptual ability to obtain an overview. In more detail, the decrease at T2 was larger for drivers with a lower education. Although the association between education and Raven’s score is nothing new, the buffering role against a decrease in perceptual speed is coherent with the idea that education represents a proxy of cognitive reserve (CR; [54,55]). 

The current study has some practical implications. The first issue concerns sensitivity to detect changes (i.e., age-related decline) in older adults’ driving-related cognitive abilities using repeated assessments over time. Our results showed that older adults’ performance at T2 was likely influenced by practice effects, at least in some tests. Notably, failure to account for practice effects in longitudinal assessments can result in incorrect inferences about changes in cognitive abilities over time [42]. For instance, practice effects could mask cognitive decline and lead the researcher to draw incorrect conclusions in favor of stability or minor changes. For this reason, future studies are needed to estimate the magnitude of practice effects on test–retest performances at test batteries such as the VTS. Moreover, it has been suggested that the development of alternate forms could provide a method to minimize the influence of practice effects [56].

A second issue concerns re-evaluation over time. A better understanding of the longitudinal trajectories of driving-related cognitive functions could provide practitioners with information to time follow-up assessments. Notably, the costs of cognitive assessments of fitness to drive contribute to healthcare spending and can be at the patient’s expense. Our results showed a decline in selective attention and inductive reasoning at T2; moreover, the decrease in selective attention was larger for drivers who were reevaluated after a longer period of time. Finally, the length of the interval between T1 and T2 assessments was negatively associated with reaction time under stress and inductive reasoning. These findings seem to support the need for reevaluations and monitoring of driving-related cognitive abilities, suggesting that assessments should occur at regular intervals of time. 

A third implication concerns restricted drivers. Behavioral restrictions are generally imposed by licensing authorities to reduce crash risk for older drivers with some functional impairment by limiting the drivers’ exposure to high-risk situations (e.g., driving at night or long distances). Existing research has shown that restricted drivers take fewer trips, drive slower, and drive shorter distances [27]. However, the restricted drivers’ crash rates still exceeded those of control drivers [27]. Previous studies also found that reduced driving practice (i.e., less than 3000 km per year) is a predictor of lower driving fitness [16,17,18]. This relationship could be explained by the fact that older drivers with limited cognitive abilities may modify their driving (e.g., driving less) either because of imposed restrictions or self-limitation [57]. Consistent with this evidence, in our results, restricted drivers obtained poorer performances than unrestricted drivers at T2 assessment, and this difference concerned almost all of the cognitive functions tested. Overall, restricted drivers may thus be a target population for training interventions to improve driving-related cognitive skills and possibly compensate for impairments [58,59,60].

Some limitations bear noting. A first limit of the study concerns the sample. The sample size was relatively small, and data were derived from the database of a psychodiagnostic service rather than from random sampling. Other limitations concerned our sample. Most participants were male, the two groups of restricted and unrestricted drivers had different sample size, and, finally, data about some tests were not available at T2. Notably, however, our results are consistent with those of previous studies [39,40]. In addition, prior literature has shown that women tend to stop driving earlier in later life (e.g., [60]) and this may partly account for the gender imbalance in our sample.

A second limitation is that the test battery used in the study included a limited number of tests. In addition, although the VTS is a widely employed instrument to measure driving-related abilities, scant evidence exists concerning its predictive validity (i.e., associations between test scores and driving performance) in older samples. Nonetheless, in a previous cross-sectional study, the performance obtained at the VTS tests was significantly associated with some aspects of driving behavior, such as having some restriction imposed by the authorities and self-limited driving.

Third, we could not provide data about a second follow-up (T3) due to the low sample size, thus limiting the chance to understand age-related changes in driving-related cognitive abilities over longer periods of time. Moreover, prior studies [40,42] suggest that shorter retest intervals are more susceptible to practice effects and the interval between the first and second assessments is likely to have the largest practice effects. Future prospective studies could address this issue analyzing older adults’ driving-related cognitive performance in at least three repeated assessments.

Finally, our study did not include a measure of driving competence. Although some information regarding driving behavior (e.g., number of accidents and fines) was collected during the clinical interview, these data consist of self-reported information. The results of previous research seem to suggest similar longitudinal trajectories for cognitive and driving performance in later life [39]; however, future prospective studies could further investigate age-related changes in both these domains over time.

## 5. Conclusions

The main goal of the current study was to examine whether the driving-related cognitive abilities of older drivers referred for cognitive assessment change over a period of time, ranging from one to four years. In more detail, we examined whether older drivers’ performances on the standard test battery of the VTS declined from the first to the second administration. Our results showed that most cognitive abilities remained stable over the short-term. However, significant decreases in selective attention and inductive reasoning suggest the need to monitor and re-evaluate driving-related cognitive abilities over time. Moreover, the results showed improvements in processing speed consistent with practice effects. Future studies are needed to estimate the magnitude of practice effects on test batteries such as the VTS to avoid incorrect conclusions about the longitudinal trajectories of older drivers’ cognitive functioning.

Finally, restricted drivers also obtained significantly poorer performances than unrestricted drivers at the T2 assessment. We thus suggest that restricted drivers may be a target population for training interventions to improve driving-related cognitive skills and to possibly compensate for impairments.

## Figures and Tables

**Table 1:** Descriptive statistics (means and standard deviations) and T scores.

	Time 1				Time 2			
	Restricted		Unrestricted		Restricted		Unrestricted	
Measure	M *(SD)*	T *(SD)*	M *(SD)*	T *(SD)*	M *(SD)*	T *(SD)*	M *(SD)*	T *(SD)*
Reaction Time								
Decision time (msec)	736 *(214)*	42 *(8)*	566 *(140)*	51 *(9)*	657 *(233)*	46 *(9)*	545 *(148)*	54 *(12)*
Decision time variability (msec)	146 *(81)*	46 *(9)*	119 *(78)*	51 *(11)*	133 *(68)*	47 *(11)*	89 *(35)*	55 *(11)*
Motor speed (msec)	306 *(93)*	47 *(8)*	265 *(87)*	51 *(11)*	298 *(93)*	47 *(9)*	256 *(58)*	51 *(7)*
Reaction Time under Stress								
Correct responses	105 *(33)*	44 *(10)*	134 *(34)*	51 *(7)*	104 *(36)*	44 *(10)*	122 *(44)*	48 *(9)*
Selective Attention								
Time correct rejection	6.17 *(2.71)*	36 *(10)*	4.36 *(1.67)*	49 *(14)*	6.09 *(2.58)*	33 *(12)*	5.00 *(2.83)*	43 *(12)*
Obtaining an overview								
Parameter	−1.64 *(0.97)*	-	−0.71 *(1.19)*	*-*	−1.66 *(1.05)*	-	−1.42 *(0.99)*	-
Inductive reasoning	26.83 *(4.79)*	-	30.29 *(4.16)*	-	24.31 *(5.38)*	-	28.69 *(4.85)*	-

**Table 2:** Repeated measure ANCOVA results. Significant effects (*p* < 0.05) are in bold, marginally significant effects (*p* < 0.10) in italics.

Measure	*F*	df	df_error_	*p*	η^2^
*Decision Time*					
Time	7.57	1	54	**0.008**	0.123
Time × Restrictions	0.32	1	54	0.572	0.006
Restrictions	13.25	1	54	**0.001**	0.197
Age	0.29	1	51	0.591	0.006
Education	1.10	1	51	0.299	0.021
Interval T1–T2	0.35	1	51	0.553	0.007
Time × Age	4.09	1	51	**0.049**	0.074
Time × Education	1.76	1	51	0.191	0.033
Time × Interval	0.34	1	51	0.560	0.007
*Decision Time Variability*					
Time	3.33	1	54	*0.074*	0.058
Time × Restrictions	1.11	1	54	0.298	0.020
Restrictions	6.73	1	54	**0.012**	0.111
Age	0.46	1	51	0.500	0.009
Education	0.13	1	51	0.716	0.003
Interval T1–T2	1.45	1	51	0.233	0.028
Time × Age	4.37	1	51	**0.042**	0.079
Time × Education	6.39	1	51	**0.015**	0.111
Time × Interval	0.70	1	51	0.407	0.014
*Motor Time*					
Time	0.02	1	54	0.890	0.000
Time × Restrictions	0.10	1	54	0.748	0.000
Restrictions	3.76	1	54	*0.058*	0.065
Age	0.03	1	51	0.873	0.001
Education	0.76	1	51	0.387	0.015
Interval T1–T2	0.39	1	51	0.534	0.008
Time × Age	0.87	1	51	0.356	0.017
Time × Education	0.80	1	51	0.374	0.016
Time × Interval	0.18	1	51	0.779	0.002
*Reaction Time under Stress*					
Time	1.79	1	51	0.187	0.034
Time × Restrictions	1.79	1	51	0.187	0.034
Restrictions	4.15	1	51	**0.047**	0.075
Age	8.41	1	48	**0.006**	0.149
Education	0.66	1	48	0.420	0.014
Interval T1–T2	4.22	1	48	**0.045**	0.081
Time × Age	0.12	1	48	0.734	0.002
Time × Education	0.04	1	48	0.846	0.001
Time × Interval	1.08	1	48	0.304	0.022
*Selective Attention*					
Time	12.63	1	44	**0.001**	0.223
Time × Restrictions	2.25	1	44	0.141	0.049
Restrictions	11.98	1	44	**0.001**	0.214
Age	3.04	1	41	*0.089*	0.069
Education	0.00	1	41	0.955	0.000
Interval T1–T2	0.01	1	41	0.937	0.000
Time × Age	0.14	1	41	0.713	0.003
Time × Education	0.23	1	41	0.635	0.006
Time × Interval	4.10	1	41	**0.050**	0.091
*Perceptual Overview*					
Time	3.16	1	56	*0.081*	0.053
Time × Restrictions	3.11	1	56	*0.097*	0.048
Restrictions	7.89	1	56	**0.008**	0.120
Age	0.89	1	53	0.351	0.016
Education	1.48	1	53	0.230	0.027
Interval T1–T2	0.00	1	53	0.957	0.000
Time × Age	0.04	1	53	0.853	0.001
Time × Education	4.52	1	53	**0.038**	0.079
Time × Interval	0.09	1	53	0.771	0.002
*Inductive Reasoning*					
Time	4.57	1	43	**0.038**	0.096
Time × Restrictions	0.14	1	43	0.706	0.003
Restrictions	8.11	1	43	**0.007**	0.159
Age	1.77	1	40	0.191	0.042
Education	6.82	1	40	**0.013**	0.146
Interval T1–T2	13.53	1	40	**0.001**	0.253
Time × Age	0.69	1	40	0.410	0.017
Time × Education	2.36	1	40	0.132	0.056
Time × Interval	1.11	1	40	0.298	0.027

## Data Availability

Data are available upon request to the first author.
